# Finding Balance: Insights from Mexican Heritage Families on Living a Healthy Lifestyle in New York City

**DOI:** 10.1007/s10900-026-01607-5

**Published:** 2026-07-23

**Authors:** Jennifer Leng, Linda Ivette Gonzalez-Sarinana, Stephanie Minaya, Bharat Narang, Jaime Gilliland, Francesca Gany

**Affiliations:** 1 Immigrant Health and Cancer Disparities Center, Department of Psychiatry and Behavioral Sciences, Memorial Sloan Kettering Cancer Center, 633 Third Avenue, 4th Floor, New York, NY 10017 USA; 2 Department of Population Health Sciences, Weill Cornell Medical College, New York, NY USA; 3 Department of Psychiatry and Behavioral Sciences, Memorial Sloan Kettering Cancer Center, New York, NY USA; 4 Department of Medicine, Memorial Sloan Kettering Cancer Center, New York, NY USA; 5 Department of Medicine, Weill Cornell Medical College, New York, NY USA

**Keywords:** Obesity, Hispanic or Latino, Health Behavior, Diet, Exercise, Family

## Abstract

Obesity disproportionately affects Hispanic adults and children, especially Mexican Americans. Family-centered interventions (FCIs) for weight loss and obesity prevention tailored to these populations are lacking. Our study (*ClinicalTrials.gov* NCT07144800) aims to develop a multicomponent culturally tailored FCI for Hispanic American families. To inform intervention development, we conducted 6 Spanish language focus groups with 31 parents/caregivers with overweight or obesity who were recruited from a Hispanic community-serving health services program in New York City. Using rapid qualitative data analysis of focus group content, 4 themes emerged: (1) *Navigating a mixture of cultures*: children were more rapidly acculturating to U.S. norms, resulting in tensions over food choices; (2) *Family functioning and parenting styles*: parents saw themselves as leaders/role models, balancing guidance with flexibility around children’s preferences; (3) *Challenges to maintaining healthy habits*: maintaining healthy diets and regular physical activity for the family was influenced by internal factors (limited motivation, low prioritization) and external pressures (work hours/time, limited financial resources); and (4) *Building on an existing foundation of knowledge*: participants had knowledge and awareness of the importance of a healthy lifestyle, which they were eager to build on with additional education and peer support. Intervention refinements should focus on leveraging authoritative parenting styles and helping families establish rules and routines around healthy habits, including nutritious and culturally familiar meal planning, and navigating the acculturation gap. Information and resources on low-cost food options and low/no-cost opportunities for physical activity should be provided.

## Introduction

Hispanics constitute the largest minoritized group in the United States (U.S.) and are projected to continue growing [1]. Mexicans represent the largest Hispanic subgroup, accounting for approximately 60% of the Hispanic population [2] and nearly 22% of all immigrants in the U.S [3]. Obesity disproportionately affects the Hispanic population. An estimated 82.7% of Hispanic adults, and 84.9% of Hispanic adults of Mexican origin, are overweight or obese (BMI ≥ 25.0 kg/m^2^), compared to 70.5% of non-Hispanic White adults [4]. Obesity also is more prevalent among Hispanic children, particularly those of Mexican origin, than among non-Hispanic White children. Among Mexican American girls and boys, 24.9% and 29.2%, respectively, are obese compared to 14.8% and 17.4% of non-Hispanic White girls and boys, respectively [5]. Parental obesity is a strong predictor of childhood overweight and obesity [6–9], and it can more than double the risk of future adult obesity among obese and nonobese children under age 10 [10]. This increased risk is partly due to genetics but also due to parent modeling of health behaviors and the home environment; risk is even greater among low-income and underserved communities [11].

Efforts to address obesity must target both adults and children [11]. Family-centered interventions (FCIs) leverage Family Systems Theory (FST), in which mutually influencing interactions within the family can support and model healthy behavior [12–21]. FCIs may lead to greater and more sustainable change, and are considered the “gold standard” for childhood obesity treatment [12–21]. An authoritative parenting style, that includes moderate control and monitoring of children by parents and shared family decision making [22], has been found to increase the likelihood of health-improving behaviors in FST-based interventions [14, 22]. A meta-analysis of FCIs targeting childhood obesity showed both short and long-term change in children’s BMI among the majority of studies, although only 1 of the 20 studies included Hispanics [12]. Recent systematic and scoping reviews revealed there is a dearth of literature describing culturally tailored FCIs for Hispanics [11, 12, 23, 24]. FCIs that jointly target both adult and child outcomes in tandem are limited (most focus on child outcomes), even though parent weight loss is a significant predictor of child weight loss [17, 23, 25, 26]. Although parent involvement appears to be crucial [24, 27], the optimal combination of parent- and/or child-focused intervention components is unknown [28].

In our prior work to address adult obesity in the NYC Mexican community, focus group participants were particularly concerned about obesity in their children [29]. They described family as a primary motivator for behavior change, they expressed a desire for group-based motivation, and they noted that time constraints were barriers to intervention adoption [29]. Building on these findings, we developed Family COMIDA (Consumo de Opciones Más Ideales De Alimentos) (Eating More Ideal Food Options), a multiphase study grounded in Social Cognitive Theory and Family Systems Theory (FST), that aims to identify the most effective FCI for weight loss and obesity prevention for Hispanic families in the US. In this article, we describe the results of Phase 1, in which we conducted Spanish language focus groups with Hispanic parents/caregivers with overweight or obesity to refine and inform additional cultural tailoring of our family-centered intervention components developed in our prior work.

## Methods

This study was reviewed and approved by the Institutional Review Board at Memorial Sloan Kettering Cancer Center (MSK) (MSK protocol #25 − 003). The Family COMIDA clinical trial is registered at ClinicalTrials.gov (NCT07144800). From August through October 2025, we conducted focus groups with parents or primary caregivers to inform refinement of planned study materials, i.e. additional cultural tailoring, and inclusion of FST concepts to (a) the baseline package component of written/digital diet and physical activity (PA) materials, self-monitoring tools, and diet/PA/text messages; and (b) the 4 experimental components (delivered via videoconference, e.g., Zoom): (1) Parent Initial Counseling; (2) Child Education; (3) Parent Weekly Individual Telephone Support; and (4) Parent Monthly Group Support.

### Recruitment

Participants were recruited through convenience sampling and first approached by a bilingual (Spanish/English) clinical research coordinator (CRC) (LIGS, SM) in-person at the “Ventanillas de Salud” (VDS) (“Health Windows”) program at the Mexican Consulate in New York City (NYC). The VDS is a health promotion collaboration between the Mexican government and over 400 U.S. non-profit and private agencies for low-income U.S. Hispanics; the program aims to address health disparities related to access to care and socioeconomic determinants, and now has 50 sites at Mexican Consulates throughout the U.S. The NYC VDS is a partnership led by the MSK Immigrant Health and Cancer Disparities (IHCD) Center and the NYC Mexican Consulate. CRCs administered a brief screening tool to review focus group eligibility criteria, and eligible participants provided verbal consent and were registered to the study. CRCs then scheduled participants for a focus group. Participants were offered a $30 gift card upon completion of the focus group.

Participant inclusion criteria included: (1) age ≥ 18; (2) self-identifies as Hispanic/Latino; (3) seeking, or has used, VDS services; (4) is the parent or primary caregiver for at least one child aged 8–12 living in their household; (5) screens as obese (BMI > 30 kg/m2) or overweight (BMI of 25–29.9 kg/m2); (6) owns a cell phone capable of receiving text messages; and (7) owns an internet-connected device capable of conducting videoconference calls. Exclusion criteria included (1) pregnant or might be pregnant; (2) lactating; (3) presence of a chronic disease, such as cancer, kidney disease, liver disease, etc. (individuals with hypertension or diabetes may still participate); (4) has dietary restrictions (i.e. liquid diet); (5) has a household member who has already participated (or agreed to participate); and (6) prior participation in one of our preliminary studies.

### Data Collection

Focus groups were conducted in Spanish, in-person or via videoconference (*Zoom*, San Jose, CA) by a Senior Program Manager trained in qualitative research methodology (JG, CGL). Focus groups lasted 90–120 min and followed a semi-structured facilitation guide developed by study team members with content (JL) and methodological (JLG) expertise, according to established methodologic guidelines [30]. The facilitation guide was organized into the following sections: (1) Introduction and participant opinions on overweight and obesity, diet, physical inactivity, and associated health risks among adults and children; (2) Participant descriptions of family diet and physical activity habits; (3) Participant opinions on additional cultural tailoring and incorporation of FST into the Family COMIDA program components; (4) Participant input on how best to structure delivery of program components; and (5) Participant-identified barriers and facilitators to program success. The guide was first created in English and then translated into Spanish. One translator conducted an initial translation, and a second provided a quality assurance review (i.e. for accuracy, style, register). The two translators then met to reconcile any differences, and then provided a signed Certificate of Authenticity attesting to the accuracy of the translation. Focus groups were audio recorded, transcribed, and de-identified for analysis.

### Data Analysis

The analytic team consisted of a qualitative methods specialist (QMS; JG), the PI (JL), and two bilingual CRCs (LIGS, SM). The QMS first trained analytic team members on best practices for rapid qualitative data analysis (RA) [31, 32]. CRCs used focus group audio recordings and transcripts to prepare one summary, in English, for each focus group. Summaries were organized in accordance with facilitation guide sections and included verbatim quotations of illustrative statements. Once all summaries were complete, they were organized into a data matrix. Members of the analytic team then independently reviewed the statements grouped within each facilitation guide section, across all focus groups, to identify concepts of interest. During a series of consensus meetings, team members then collaboratively reviewed findings organized in the data matrix to identify and refine recurrent concepts of interest and to synthesize thematic findings [33].

## Results

We approached 95 individuals attending the VDS; 50 were ineligible, and 45 were eligible, of whom 31 agreed to participate in a series of 6 focus groups. Reasons for not participating included not having enough time to participate, scheduling conflicts (not being available at time of focus group), or not being interested.

Participant characteristics are shown in Table 1. All participants were female (*n* = 31, 100%). The mean age was 40.6 years (6.8 SD), and 25 (86.2%) reporting having limited English proficiency (speaking English less than “very well”) (2 missing). Most (*n* = 28, 90.3%) were born in Mexico, and 1 was born in the U.S. (2 missing). The mean number of years living in the U.S. was 19.75 (7.6 SD; range, 2–31 years). Most were married (*n* = 12, 41.4%) or partnered (*n* = 8, 27.6%); the rest were divorced (*n* = 1, 3.4%), widowed (*n* = 2, 6.9%), or single (*n* = 6, 20.7%) (2 missing). Two had no formal schooling (6.9%), and 1 had less than a primary school education (3.4%); 11 had completed primary school (37.9%), 13 (44.8%) secondary/high school, and 2 (6.9%) had some college; none were college graduates and 2 responses were missing. Participants had a range of health coverage types (Emergency Medicaid only, *n* = 4 [19%}; Medicaid HMO, *n* = 3 [14.3%]; Private PPO, *n* = 1 [4.8%]; NYC Care only, *n* = 11 [52.4%]; NYC Care + Emergency Medicaid, *n* = 1 [4.8%]; other, *n* = 1 [4.8%]); 8 participants (38.1%) had no health coverage, and 2 were missing. Twenty participants were currently working (69%) (2 missing), 12 in cleaning/maintenance, and 1 each in factory/warehouse work, waitering/bartending, laundry/dry cleaning, sales work, health care, secretarial work, and nail technician work. Mean self-reported approximate monthly household income was $2773.9 (1635.2 SD; range, $500-$6400).

**Table 1:** Participant Characteristics (*n* = 31)

Characteristic	Group	No. (%) / Mean (SD) [range]
Gender	Female	31 (100%)
	Male	0
Age, y		40.6 (6.8) [26–59]
Spoken English Proficiency	English Proficient Limited English Proficiency	4 (13.8%) 25 (86.2%)
	Missing	2
Years in the U.S.		19.75 (7.57) [2–31]
Place of birth	Mexican State/City	
	Puebla	12 (41%)
	Guerrero	6 (20.7%)
	Jalisco	2 (6.9%)
	Mexico City	1 (3.4%)
	Hidalgo	1 (3.4%)
	Morelos	1 (3.4%)
	Oaxaca	1 (3.4%)
	Tlaxcala	1 (3.4%)
	Veracruz	1 (3.4%)
	State unspecified	2 (6.9%)
	United States	1 (3.4%)
	Missing	2
Marital Status	Married Partnered Divorced Widowed Single Missing	12 (41.4%) 8 (27.6%) 1 (3.4%) 2 (6.9%) 6 (20.7%) 2
Education	No formal schooling	2 (6.9%)
	Less than primary school	1 (3.4%)
	Primary school completed Secondary or high school graduate or GED	11 (37.9%) 13 (44.8%)
	Some college	2 (6.9%)
	College graduate Missing	0 2
Health insurance	Emergency Medicaid only	4 (19%)
	Medicaid HMO	3 (14.3%)
	Private PPO	1 (4.8%)
	Coverage at New York Health + Hospitals through NYC Care Program	11 (52.4%)
	Emergency Medicaid + NYC Care Other No health insurance Missing	1 (4.8%) 1 (4.8%) 8 (38.1%) 2
Employment status	Currently working	20 (69%)
	Unemployed/not working Missing	9 (31%) 2
Occupation	Cleaning/maintenance	12 (48.1%)
	Factory/warehouse Waiter/Bartender Laundry/Dry cleaner Sales Health Care Secretary Nail tech	1 (5%) 1 (5%) 1 (5%) 2 (10%) 1 (5%) 1 (5%) 1 (5%)
Approximate Household Monthly Income		2773.9 (1635.2) [500–6400]

We identified four key themes centered around how participants organize their lives related to diet and physical activity, and how these can be used to inform Family COMIDA intervention content and delivery: (1) Navigating a mixture of cultures, (2) Family functioning and parenting styles, (3) Challenges to maintaining healthy habits – internal and external factors, and (4) Building on an existing foundation of knowledge. Themes and subthemes are described below, with additional detailed illustrative quotations in Table 2.

**Table 2:** Themes/subthemes and additional illustrative quotations

Themes and sub-themes	Illustrative Quotations
Theme 1. Navigating a mixture of cultures	
Acculturation	“Because of how we live now with stress, work, appointments … I often just buy them fast food. Living and working in the U.S. makes it easier to buy food from restaurants.”
	“On Sundays, I give them a day off so they can eat fast food like McDonald’s… they need to be like other children and try everything.”
	“Sometimes we try to include vegetables and healthy foods, but our kids have grown up used to American food and they prefer McDonald’s or other fast food.”
	“Some of the things that contribute to overweight in this country are stress and having less time to eat properly. For kids, lack of exercise is a major factor.”
Cultural background	“In my case, we cook Mexican food that’s often spicy, so our kids don’t want to eat vegetables. They eat fruit, but not vegetables, so their diet isn’t balanced.”
***Theme 2: Family functioning and parenting styles***	
Decision making around food	“I am the chef, so I decide what we usually eat…like this weekend I told them we were going to eat salmon or something healthy…sometimes I ask my husband if he wants something.”
	“My husband and I make the decisions together. When I cook, I decide, but when he’s not working and cooks, he decides. Sometimes we consider our kids’ opinions.”
	“I decide what’s good and not good for me and my family.”
	“(it’s up to me) to eat healthier, cook better meals, not as fatty. I am the leader of the family.”
	“If we set our minds to start a routine tomorrow…we can change to be healthier. My husband couldn’t care less…it will be me.”
	“As mothers we are trying to change the menu at home…illness comes from a bad diet, and living a healthy life requires changes made by the whole family.”
Physical activity and role modeling	“Physical activity is great… it helps with our health and also brings the family together.”
	“It’s important to be part of our children’s lives, physically by playing with them, emotionally by talking to them.”
	“I think we all need to exercise… even 30-minute workouts, going for a walk, or dancing with the kids. We need to set an example by doing physical activity with them, so they’re more likely to do it too. We should look for fun and friendly activities that kids enjoy.”
***Theme 3: Challenges to maintaining healthy habits - internal and external factors***	
Internal factors – limited motivation and low prioritization	“We just don’t take care of ourselves…we focus on our kids and we as parents do not focus on ourselves.”
	“We fall into a [unhealthy] routine… but we [could] organize ourselves to take the children on a morning walk.”
	“We focus too much on work ….so it’s easier to buy things on the road…we don’t take the time [to cook and exercise], that’s why we don’t notice until your pants don’t close.”
	“We are not disciplined to have a healthy diet…it is easy to go the guy on the corner.”
External factors – time and financial limitations, limited outdoor space, screens	“I am a stay home mom and between cleaning and tending for the kids, I don’t have enough time to exercise.”
	“Kids spend too much time on the internet and on their iPads. They need to exercise instead.”
	“I try to make time to take him to the park. Can move for free, can have that physical activity.”
	“If a person has a good socioeconomic level, they can have more access to fast food…it saves you a lot of time.”
***Theme 4: Building on an existing foundation of knowledge***	
Knowledge and awareness of the importance of a healthy lifestyle	“Obesity in adults is caused by the food we eat… in my case we need to change the way we eat, because we are concerned about the health issues this [our eating habits] might bring… my husband mentioned he wants to eat healthier, more vegetables, without any fat.”
	“It’s the parents’ fault that our kids are obese. If we don’t take care of ourselves, it’s harder to ask our kids to take care of themselves. We have to give them healthy food. Giving them junk like candy, we’re causing them to develop diseases, just like adults.”
	“We [parents] need to help out and make sure our children [are] eating the appropriate portions as bad nutrition is one of the leading problems.”
	“Teenagers often have low self-esteem because of being overweight. My daughter feels embarrassed about her weight and keeps her sweater on all the time…at school, they can be bullied.”
Desire for change, additional information/ education, and facilitators to program success	“When we come to this country, the food changes. In Mexico, our culture doesn’t teach us to eat healthily. Seeing that here we’re taught and even rewarded for it, that makes me very happy.”
	“I think [the Family COMIDA program sounds] great. It [will] educate us and help us develop healthier habits. I’d love to participate with my kids, especially my 5-year-old who struggles with gaining weight. This program would be great for my husband and the whole family.”
	“It’s a great help because we make so many excuses—like not having time or not knowing what’s healthier. Learning what to avoid will really help us build healthier habits.”
	“I think it’s great [if] somebody else tells them [our children] and teaches them how to eat healthy… because sometimes they don’t know and think it’s us being mean [to them].”
	“I think it’s great because kids are very curious and want to help in the kitchen, so learning safe ways to cook could be great.”
	“The kids do tell me the nutrition tips they learn from school, so they will definitely absorb this information [from Family COMIDA].”
	“It would be a challenge [to eat healthier] for the whole family because food is getting more expensive every day, and it’s hard to eat healthy. But it’s worth it for a longer and healthier life. A [program] like [Family COMIDA can] help us work towards a healthier adulthood.”

### Theme 1. Navigating a Mixture of Cultures

#### Acculturation

Across focus groups, participants described navigating their own process of cultural transition in the U.S. while raising children who were more rapidly acculturating to U.S. norms. Specifically, tensions surrounding food practices emphasized how parents’ traditional Mexican dietary patterns often conflicted with American food preferences adopted by their children:

“*My kids are more used to eating fast food like Chinese or pizza. I try to save that kind of food for weekends. They still eat our Mexican food*,* but they prefer fast food.”*

Parents described food tensions as a generational divide, given their children’s exposure to a broad range of American food options:

“*Raised in the United States…everyone likes something else*,* there’s some division between parents’ and children’s eating habits*”.

While some parents acknowledged that, in addition to children’s preferences for fast food, it was also increasingly consumed for convenience, given the fast-paced life in the U.S., time constraints, work schedules, stress, and affordability:

“*Fast food is a quick fix*,* and sometimes even cheaper than cooking…a pizza can cost less than buying and preparing a whole chicken…vegetables are especially expensive*,* especially for a big family*.”

Other parents stated that fast food was too expensive and unhealthy:

*We don’t consume fast food – we can’t afford to*,* and I prefer to cook healthier meals*.

Parents also described the busy U.S. lifestyle and long school hours contributing to reduced physical activity:

*In Mexico*,* you finish eating and go out to play. Here you can’t do that.*

#### Cultural Background

Participants described cultural norms as playing an important role in food consumption, including the belief that a “bigger plate” of pasta or rice provides better value, even if it is less healthy; and using traditional cooking methods that include high amounts of fat and sodium (e.g. in tacos, tamales, pozole, chilaquiles, enchiladas). Parents were concerned about a lack of nutrition education and about the generational nature of unhealthy eating and it being passed down:

*We often don’t know what to eat to avoid becoming overweight…I was diagnosed with diabetes…it wasn’t until then that I looked for help and tried to change my lifestyle and my family’s eating habits*.

*Our parents taught us to eat this way*,* not in a balanced or healthy way*.

Parents described efforts to establish healthy eating practices while preserving traditional food customs, such as reducing carbohydrates, using less cooking oil, reducing portion sizes, and including fruits and vegetables in meals, although some mentioned that the spiciness of Mexican food was sometimes a barrier to their children eating vegetables.

### Theme 2. Family Functioning and Parenting Styles

#### Decision-making Around Food

Participants described a range of approaches to food-related decision-making within their households. Nearly all participants (all were mothers) described being primarily responsible for grocery shopping and cooking. Most adopted an authoritative approach, by taking control over meal decisions, while some explained they had a more collaborative approach, seeking input from family members when planning meals, and some fell between authoritative and collaborative styles, depending on the situation:

*They have to eat what I cook… the younger one sometimes won’t eat*,* but after 10 min*,* she will*.

“*I decide what they eat because I do the shopping and the cooking. I choose what goes on the table.”*

*I ask for opinions on what they want to eat… if it’s healthy and I can afford it; I let them buy it*.

Some participants stated that as mothers they were the leaders of the family and reported a strong sense of responsibility for modeling behaviors for their children, balancing guidance with flexibility. Participants reported valuing routines but applying them adaptively, striving to avoid being punitive or authoritarian:

“*As a family*,* we should talk about what we all enjoy*,* so we can agree on something healthy for everyone…it’s harder to get younger kids to eat vegetables*” (Participant 4, Focus Group 5).

*I’m not strict…I don’t like to punish my kids with food*.

#### Physical Activity and Role Modeling

Several parents noted that children primarily engaged in physical activity through school sports, as opportunities for physical activity at home were often limited. They shared that they themselves had only recently begun exercising, often because of health concerns, not as a preventive habit:

*My kids usually exercise at school. My husband and I started exercising because of health problems*,* that’s what finally motivated us to do it*.

Some parents mentioned being physically active through physically demanding jobs or through errands/daily routines. Others emphasized their role as models, understanding that their lifestyle habits strongly influenced their children’s behaviors:

*It’s important to recognize how [certain] bad habits are influenced by the role of the parent within the household.*

Despite work schedules and other time constraints limiting opportunities for structured exercise *(“…our schedules make it hard for us*,* varying shifts and only Saturday off*,* but that day is just for chores.*”), some participants described integrating movement into family life through going to the park together, active play, and doing calisthenics at home. Some participants viewed physical activity as an essential way to strengthen family bonds, in addition to supporting physical and mental well-being.

### Theme 3. Challenges to Maintaining Healthy Habits – Internal and External factors

#### Internal Factors

Participants cited limited motivation and low prioritization of healthy habits as complicating factors:

*Sometimes as Latinos we fall so much into the work-home/work-home routine that we feel tired and don’t take the time to exercise*.

Others described difficulty breaking sedentary habits:

…*people feel comfortable*,* they get stuck…not that they can’t…(they) just don’t make the effort to do it*.

#### External Factors

Time constraints due to long work hours and financial limitations were seen as the most influential external pressures (overlapping with subthemes discussed above related to acculturation and physical activity/role modeling). Lack of time influenced family habits and routines around meal preparation and physical activity. Participants’ long workdays often resulted in them choosing fast or convenience foods. Routines and choices were complicated by cost, as some participants noted convenience foods are often a better value:

*As a mother*,* I know that this [meals served] contributes to my child’s weight gain… sometimes junk food is cheaper than healthy food*.

Cost barriers also limited access to gyms and structured physical activity, resulting in reliance on school activities to meet children’s physical activity needs and reliance on daily routines (e.g., walking as part of their commute, physically demanding jobs) for adults:

*The type of activities my kids want are expensive… if I had the economic access*,* I would be interested in these activities*.

Participants also compared the more active lifestyles they experienced in Mexico with the restrictions of life in the U.S., particularly in the urban setting with limited outdoor space and concerns about neighborhood safety. Further, screens were noted both as a distraction for adults and as a means to keep children occupied:

*It’s hard [to exercise] because of my cellphone use*.

*We work*,* so we give the phone to our kids to entertain them*,* gives them something to do*.

### Theme 4. Building on an Existing Foundation of Knowledge

#### Knowledge and Awareness of the Importance of a Healthy Lifestyle

Participants demonstrated a strong understanding of what constitutes a healthy lifestyle, recognizing the importance of a nutritious diet and physical activity to avoid obesity and associated chronic conditions:

*Obesity in adults can lead to disease such as diabetes and high cholesterol*,* due to not taking care of their diet and not exercising*.

*In my opinion*,* it’s about eating healthy and taking care of yourself…avoid street food*,* eat vegetables*,* drink water…not just for myself*,* but for my whole family*.

Parents were also aware of the benefits of a healthy weight and exercise for their children, in particular to boost confidence and avoid bullying:

*The main benefit [of exercise] is mental and physical health…it makes them feel more self-confident because they are not exposed to being teased by other children*.

#### Desire for change, additional information/education, and facilitators to program success

Participants expressed a strong desire to make healthy changes to their families’ diet and exercise habits, through engaging in physical activity together, setting clear but flexible boundaries around screen time, and finding ways to shape healthy food choices to best fit their family dynamics:

*We as adults need to do our part—we have to work on our eating habits. No one else is going to come and make the changes we need to make. We have to educate ourselves and take action*.

Participants reported that a Family COMIDA intervention that provided further nutrition and physical activity education, peer support, and information on community-based resources would help enable them to develop realistic, sustainable routines, building on their existing knowledge gained through attending informational sessions at schools and community organizations. Parents emphasized the importance of family discussions and shared commitments to support lasting behavior change. They valued receiving clear, practical nutrition information, particularly guidance on healthy food choices, meal planning, simple recipes, and home-based exercise ideas:

“*All of this [this focus group] has been great for me. I really like hearing other perspectives. I appreciate this support because*,* as Mexicans*,* we don’t know how to eat properly*,* don’t really have a foundation for combining foods to create a healthy meal*.”

*I think [Family COMIDA sounds] great*,* it would help me with menus and knowing what to cook. I usually make the same three things*,* so a healthy menu would be really helpful*.

Many participants also highlighted the value of peer support, noting that connecting with other families facing similar challenges could increase motivation, accountability, and self-efficacy:

*Excited for forming a group with a common goal and having teammates*.

Participants also cited schools and pediatricians as important partners in providing credible information:

*Listening to different opinions helps a lot… every mom is struggling… I like to get informed. Usually*,* I get my information from school or the pediatrician*.

Due to time constraints, there was general consensus among participants that a virtual format would be more convenient and accessible for education sessions for both adults and children. Flexibility in the timing and scheduling of education sessions was also identified as a key facilitator, with participants stating that offering multiple session options outside of the standard 9 − 5 work schedule would facilitate attendance.

## Discussion

This study aimed to gather evidence to refine and further culturally tailor the Family COMIDA program, an FCI for weight loss and obesity prevention among Hispanic families in the U.S. All Hispanic parents who participated in our focus groups expressed positive interest in the Family COMIDA program, consistent with our prior work and other examples from the literature describing community member support for culturally adapted lifestyle interventions [29, 34–37]. There were indications that an intervention like Family COMIDA, which is grounded in SCT and FST, can build on existing strengths within the target population, including a foundation of existing knowledge, authoritative parenting styles, and an openness to building new family knowledge, skills, and routines around a healthy diet and physical activity. Areas for intervention refinement emerged.

Key personal concepts in SCT are knowledge and skills, self-efficacy (individual’s belief in their ability to perform a behavior to achieve a desired outcome), and outcome expectancies (beliefs about the likelihood of a behavior resulting in a particular outcome) [38]. A desire for increased knowledge and skills was reflected in participants’ stated desire for more knowledge/education related to nutrition and building healthful meal-planning skills. Their feedback on peer support, expressing optimism about teaming up with other parents towards reaching common goals, suggested that the Family COMIDA Parent Monthly Group Support component may enhance their self-efficacy to adopt healthier habits. The presence of positive outcome expectancies was indicated in their expectations that adopting a healthier lifestyle would help them to avoid health problems, such as diabetes and high cholesterol, and deter teasing and bullying of their children.

Our participants described a desire to change their routines to establish healthier behaviors around diet and physical activity, which indicated a readiness for the approach of FST-informed interventions, which aim to help families make higher-level changes around family rules, routines and dynamics [39]. Many focus group participants in our study evidenced the authoritative parenting style that has been found to be effective in FST interventions [14, 22], often seeing themselves as the “leader” of the family but still responsive and flexible with the needs of their children and family members (avoiding punitive actions and interested in finding common ground around food choices and ways to exercise together). This indicates a need for the Family COMIDA intervention to positively reinforce authoritative parenting among parents with this style and to support other parents to develop this parenting style, as suggested by the FCI literature [39].

Participants described an acculturation gap, in which intergenerational differences related to food preferences had arisen. According to the literature, children typically assimilate more rapidly than their immigrant parents, contributing to the acculturation gap [40]. Parents frequently described preparing traditional Mexican meals, while modifying them to be healthier, and also making allowances for children to eat fast food on occasion and to “*be like other children and try everything”*. Some parents also attributed fast food consumption to its time-saving convenience and its perceived lower cost than home-cooked food. Family COMIDA will incorporate this feedback and include recipes/meal modifications that are time efficient, low cost, nutritious, and culturally familiar, while providing support and guidance on how to navigate children’s desire to eat popular American foods along with their peers.

Participant feedback on the logistics of implementing Family COMIDA was consistent with our prior work and the literature, in which time and financial constraints were key limitations to adopting healthier habits [29, 41, 42]. Participants preferred virtual formats for convenience and noted that education sessions will need to be flexibly scheduled and occur outside of usual work hours. Family COMIDA also will include low- and no-cost ideas and opportunities for physical activity to respond to the concerns raised by participants about the prohibitive costs of the physical activities in which they and their children would like to participate.

Limitations to this study include a sampling bias, given that we used convenience sampling, and that all participants were women (mothers), which limits generalizability. Despite this, as many participants stated, and as prior literature confirms [29, 43–46], mothers are frequently the main decision makers in the family related to grocery shopping and cooking. Mothers’ input is essential to informing refinements of the Family COMIDA intervention. Results from this study provide additional information on the perspectives of Hispanic parents related to an FCI focused on weight loss and obesity prevention, contributing to the limited body of knowledge on this topic in a high-risk, underserved population.

## Data Availability

Deidentified data are available upon reasonable request to the corresponding author.

## Declarations

### Editorial Support

The authors thanks Sonya J. Smyk, Memorial Sloan Kettering Cancer Center, for writing and editorial support. She was not compensated beyond her regular salary.
